# Efficacy, Retention, and Tolerability of Brivaracetam in Patients With Epileptic Encephalopathies: A Multicenter Cohort Study From Germany

**DOI:** 10.3389/fneur.2018.00569

**Published:** 2018-07-23

**Authors:** Laurent M. Willems, Astrid Bertsche, Frank Bösebeck, Frauke Hornemann, Ilka Immisch, Karl M. Klein, Susanne Knake, Rhina Kunz, Gerhard Kurlemann, Lisa Langenbruch, Gabriel Möddel, Karen Müller-Schlüter, Felix von Podewils, Philipp S. Reif, Bernhard J. Steinhoff, Isabel Steinig, Felix Rosenow, Susanne Schubert-Bast, Adam Strzelczyk

**Affiliations:** ^1^Epilepsy Center Frankfurt Rhine-Main and Department of Neurology, Goethe University Frankfurt, Frankfurt, Germany; ^2^Department of Neuropediatrics, University of Rostock, Rostock, Germany; ^3^Centre of Pediatric Research, Hospital for Children and Adolescents, Leipzig, Germany; ^4^Epilepsy Center Rotenburg, Agaplesion Diakonieklinikum Rotenburg, Rotenburg, Germany; ^5^Epilepsy Center Hessen and Department of Neurology, Philipps-University, Marburg, Germany; ^6^Epilepsy Center Greifswald and Department of Neurology, Ernst-Moritz-Arndt-University, Greifswald, Germany; ^7^Department of Neuropediatrics, Westfälische Wilhelms-University, Münster, Germany; ^8^Epilepsy Center Münster-Osnabrück, Department of Neurology with Institute of Translational Neurology - Epileptology, Westfälische Wilhelms-University, Münster, Germany; ^9^Epilepsy Center for Children, University Hospital Neuruppin, Brandenburg Medical School, Neuruppin, Germany; ^10^Kork Epilepsy Center, Kehl-Kork, Germany; ^11^Department of Neuropediatrics, Goethe University Frankfurt, Frankfurt, Germany

**Keywords:** levetiracetam, epileptic encephalopathies, epilepsy, seizure, anticonvulsants

## Abstract

**Objective:** To evaluate the efficacy and tolerability of brivaracetam (BRV) in a severely drug refractory cohort of patients with epileptic encephalopathies (EE).

**Method:** A multicenter, retrospective cohort study recruiting all patients treated with EE who began treatment with BRV in an enrolling epilepsy center between 2016 and 2017.

**Results:** Forty-four patients (27 male [61%], mean age 29 years, range 6 to 62) were treated with BRV. The retention rate was 65% at 3 months, 52% at 6 months and 41% at 12 months. A mean retention time of 5 months resulted in a cumulative exposure to BRV of 310 months. Three patients were seizure free during the baseline. At 3 months, 20 (45%, 20/44 as per intention-to-treat analysis considering all patients that started BRV including three who were seizure free during baseline) were either seizure free (*n* = 4; 9%, three of them already seizure-free at baseline) or reported at least 25% (*n* = 4; 9%) or 50% (*n* = 12; 27%) reduction in seizures. An increase in seizure frequency was reported in two (5%) patients, while there was no change in the seizure frequency of the other patients. A 50% long-term responder rate was apparent in 19 patients (43%), with two (5%) free from seizures for more than six months and in nine patients (20%, with one [2 %] free from seizures) for more than 12 months. Treatment-emergent adverse events were predominantly of psychobehavioural nature and were observed in 16%.

**Significance:** In this retrospective analysis the rate of patients with a 50% seizure reduction under BRV proofed to be similar to those seen in regulatory trials for focal epilepsies. BRV appears to be safe and relatively well tolerated in EE and might be considered in patients with psychobehavioral adverse events while on levetiracetam.

## Introduction

Brivaracetam (BRV), the second substance in the racetam class of anti-epileptic drugs (AEDs), was approved in the EU and USA in 2016 as adjunct therapy for epilepsy with focal onset seizures whether or not secondary generalization is present. Promising results concerning efficacy, tolerability and safety of BRV were demonstrated in a number of clinical trials (1–7). Like levetiracetam (LEV) BRV primarily acts as inhibitory ligand at the synaptic vesicle protein 2A (SV2A). Compared to LEV, BRV shows a 30-fold increased affinity to its structural target (8–12). Switching patients from LEV to BRV at a ratio of 10:1 to 15:1 may reduce adverse drug events in patients who respond well to LEV but develop drug-related sedation or BAEs (behavioral adverse events) (1, 7, 13).

Epileptic encephalopathies (EE) are a heterogeneous group of epilepsy syndromes (14, 15) in which epileptic activity leads to progressively greater levels of cognitive and behavioral impairment as it would be expected only as a result of the underlying structural or genetic pathology. According to ILAE guidelines (International League Against Epilepsy), common EE syndromes with characteristic electroclinical manifestations are Lennox-Gastaut Syndrome (LGS), Dravet Syndrome (DS), West Syndrome (WS) and EE with continuous spike-and-wave during sleep (CSWS). In addition, there is a heterogeneous group of diseases with metabolic, or structural aetiologies predisposing the development of EEs, such as Landau-Kleffner Syndrome (LKS), Tuberous Sclerosis Complex (TSC) or Unverricht-Lundborg Syndrome (UVR). Moreover, several epileptogenic mutations like *SCN9A, KCN2A* or *GRIN-2B* have been shown to be associated with EE in their course of disease. The majority of EE patients develop refractory epilepsies and suffer from relapsing seizures of heterogeneous semiologies. Frequent hospitalization associated with the need for extended medical and nursing care place major social, interpersonal, and economic burden on patients, caregivers and society (14–18).

The purpose of this multicenter study was to evaluate efficacy and tolerability of BRV in patients with EE.

## Patients and methods

A retrospective data analysis with EE patients who received at least one dose of BRV between 2016 and 2017 was performed at eight German epilepsy centers (Frankfurt, Greifswald, Kork, Leipzig, Marburg, Münster, Neuruppin, and Rotenburg/Wümme). There is no third party funding or sponsoring to report. This study was approved by the ethics committee. STROBE (Strengthening the Reporting of Observational Studies in Epidemiology) guidelines were followed (19).

The average seizure frequency of the last three months prior to the initiation of BRV was accepted as the baseline frequency. Three, 6 and 12 months retention rates were calculated. Terminal remission defined patients reaching seizure freedom throughout the subsequent follow up periods. For the purposes of this study, a 25% seizure reduction was assumed if seizure frequencies declined by 25 to 50% compared to the baseline whereas a 50% seizure reduction described a reduction of the seizure frequency above 50%. Patients who showed less than a 25% seizure reduction were assumed to be non-responders. A seizure increase was defined as any increase of seizure frequencies greater 25%. Further details of analysis and the definition of BAEs are available in Steinig et al. (20). Data acquisition was performed using anonymised, standardized reporting forms and statistical analysis by IBM SPSS Statistics, Version 22.0 (IBM Corp, Armonk, NY, U.S.A.). Kaplan-Meier survival curves were used to estimate retention time; Chi-Square and log-rank tests were used for statistical analysis with *p*-values < 0.05 treated as statistically significant.

## Results

### Patient characteristics at baseline

Forty-four patients (27 male; 61% male), mean age 28.8 years (±14.2, range 6-62, nine children or adolescents <18 years [21%]), were included in this study. The most frequent aetiologies of EE in our cohort were LGS (*n* = 20, 45.5%), TSC (*n* = 3, 6.8%), UVR (*n* = 2, 4.5%), and CSWS (*n* = 2, 4.5%). Moreover, several patients displayed other well-defined syndromes associated with EE, such as DS, AS and Neuronal Ceroid Lipofucinosis (each *n* = 1, 2%) or epileptogenic mutations, such as *SCN9A, KCN2A* and *GRIN-2B* (each *n* = 1, 2%). Mean epilepsy duration at baseline was 24.4 ± 15 years (median 23; range 0–57 years). Epilepsy onset was at a mean age of 4.4 ± 6.3 years (median 2; range 0–27 years). In 18 patients (40.9%), mRS (modified Rankin Scale) of 3–5 indicated moderate to severe impairment. Mean AED number at start of BRV was 2.9 ± 0.9 (median: 3, range: 1–4 AEDs). The most frequently prescribed drugs at baseline were: valproate (VPA, *n* = 27, 61%); LEV (*n* = 24, 55%); lamotrigine (LTG, *n* = 24, 55%); clobazam (CLB, *n* = 18, 41%); carbamazepine (CBZ, *n* = 14, 32%), topiramate (TPM, *n* = 14, 32%), and zonisamide (ZNS, *n* = 14, 32%). A drug refractory course was present in all patients, they have failed a mean number of 4.4 ± 4.3 AEDs in the past (median 3.5, range 0–17; current AEDs not included). A total of 37 patients (84.1%) had exposure to LEV during their lifetime. Details are presented in Table 1.

**Table 1:** Clinical and sociodemographic characteristics of the cohort (*n* = 44).

**Age (years)**	
Mean ± SD	28.3 ± 14.5
Median	26.0
Range	3–62
**Mean age at onset of epilepsy (years)**	
Mean ± SD	4.4 ± 6.2
Median	2.0
Range	0–27
**Epilepsy duration (years)**	
Mean ± SD	24.4 ± 15.0
Median	23.0
Range	0–57
**Sex**	*n* (%)
Male	27 (61.4)
Female	17 (38.6)
**Number of concomitant AEDs at start of BRV**	
Mean ± SD	2.9 ± 0.9
Median	3.0
Range	1–4
**Previously failed AEDs (without current)**	
Mean ± SD	4.4 ± 4.3
Median	3.5
Range	0–17
**Seizure semiology**	*n* (%)
Focal onset seizures with preserved awareness	3 (6.8)
Focal onset seizures with impaired awareness	20 (45.5)
Focal to bilateral tonic-clonic seizures	27 (61.4)
Myoclonic seizures	18 (40.9)
Atypical absence seizures	14 (31.8)
Other generalized seizures	20 (45.5)
**Syndrome/etiology**	*n* (%)
Lennox-Gastaut-Syndrome (LGS)	20 (45.5)
Tuberous sclerosis complex (TSC)	3 (6.8)
Unverricht-Lundborg-Syndrome (UVR)	2 (4.5)
Continuous Spike Waves in Sleep (CSWS)	2 (4.5)
Dravet-Syndrome	1 (2.3)
*SCN9A* mutation	1 (2.3)
Neuronal Ceroid Lipofuscinosis (NCL)	1 (2.3)
*KCN2A* mutation	1 (2.3)
*GRIN-2B* mutation	1 (2.3)
*RBFOXI* mutation	1 (2.3)
Angelman-Syndrome	1 (2.3)
other	10 (22.7)

*AED, anti-epileptic drug; SD, standard deviation; BRV, brivaracetam*.

The mean monthly seizure frequency during baseline period was 54.9 ± 76.9 (median 30, range 0–400). In three patients (6.8%) no seizures during baseline period were reported, however, they were switched due to TEAE (Treatment Emergent Adverse Event) under LEV or another AED. Focal onset seizures with preserved awareness were reported in three patients (7%), while 20 patients (46%) suffered from focal onset seizures with impaired awareness. 27 patients (61%) described focal onset seizures evolving into bilateral tonic-clonic seizures. Atypical absence seizures were reported by 14 (32%) and myoclonic seizures by 18 patients (41%). In addition, 20 patients (46%) described other generalized seizure types, such as tonic or atonic seizures.

### Treatment with brivaracetam

The initial daily dosage of BRV for patients who were not taking LEV at start of BRV varied between 25 mg and 100 mg (mean 66.3 mg ± 26.0 mg, median 50 mg). Median titration time was 6 days (mean 13.7 ± 15.8 days), the target dose ranged between 100 mg and 200 mg (mean 138.5 ± 50.6 mg, median 100 mg). The total number of patients switching from LEV to BRV at a median 15:1 ratio (mean 16.2:1, range 5:1 to 50:1) was 24 (55%), of whom 21 switched overnight and three overlapped LEV and BRV. Patients switched from LEV to BRV at an initial dose in the range 25 mg to 300 mg (mean 122.1 ± 72.1 mg, median 100 mg), with a target dose in the range 60 mg to 300 mg (mean 175.2 ± 70.1 mg, median 187.5 mg).

### Retention, responder rate, and seizure free patients

The probability that a patient would still be on BRV treatment after 3 months was 65%, respectively 52% after 6 months and 41% after 12 months. The most common reasons for discontinuing BRV were lack of efficacy (*n* = 12, 27%), adverse events (*n* = 5, 11%); or both (*n* = 2, 5%).

At 3 months, 20 (45%, 20/44 as per intention-to-treat analysis considering all patients that started BRV including three who were seizure free during baseline) were either seizure free (*n* = 4; 9%, three of them already seizure-free at baseline) or reported at least 25% (*n* = 4; 9%) or 50% (*n* = 12; 27%) reduction in seizures. There was no change in the frequency of seizures in 21 patients (48%), an increase in seizure frequency was reported in two (5%) patients. In one patient response was not well quantifiable. Table 2 shows the response according to clinical characteristics.

**Table 2:** Clinical characteristics and outcome on 3-months-follow-up (*n* = 43, response in 1 patients not quantifiable).

	**Patients**	**Non responders**	**>25% response**	**>50% Response**	**Subgroup of seizure free patients**
	n			% *(n)*	
Total	43	53.5 (23)	9.3 (4)	37.2 (16)	9.3 (4)
**SEX**					
Male	26	50.0 (13)	3.8 (1)	46.2 (12)	15.4 (4)
Female	17	52.6 (10)	15.8 (3)	21.1 (4)	0.0 (0)
**AGE RANGE**					
<18 years	9	44.4 (4)	11.1 (1)	44.4 (4)	22.2 (2)
≥18 years	34	55.9 (19)	11.8 (4)	32.3 (11)	5.9 (2)
**INITIAL BRV DOSAGE**					
≤ 100 mg	21	47.6 (10)	19.0 (4)	33.3 (7)	9.5 (2)
100–199 mg	14	57.1 (8)	0.0 (0)	42.9 (6)	7.1 (1)
>200 mg	5	60.0 (3)	0.0 (0)	40.0 (2)	0.2 (1)
**LEV STATUS**					
Direct switch from LEV to BRV	23	56.5 (13)	4.3 (1)	39.1 (9)	17.4 (4)
Start of BRV with previous exposure to LEV	13	61.5 (8)	7.7 (1)	30.8 (4)	0.0 (0)
Start of BRV without previous exposure to LEV	7	28.6 (2)	28.6 (2)	42.9 (3)	0.0 (0)
**PREVIOUSLY FAILED AEDs (WITHOUT CURRENT)**					
0–1	14	42.9 (6)	0.0 (0)	57.1 (8)	21.4 (3)
≥ 2	25	56.0 (14)	16.0 (4)	28.0 (7)	0.04 (1)
**NUMBER OF AEDs AT START OF BRV**					
0–1	3	66.6 (2)	0.0 (0)	33.3 (1)	33.3 (1)
2	8	62.5 (5)	0.0 (0)	37.5 (3)	25.0 (2)
≥3	28	46.4 (13)	14.3 (4)	39.3 (11)	3.6 (1)
**SEIZURE SEMIOLOGY**					
Focal onset seizures with or without preserved awareness	19	63.1 (12)	10.5 (2)	26.3 (5)	5.3 (1)
Focal to bilateral tonic-clonic seizures	26	42.3 (11)	15.4 (4)	42.3 (11)	7.7 (2)
Other generalized seizures	31	48.4 (15)	6.5 (2)	45.2 (14)	3.2 (1)

*BRV, brivaracetam; LEV, levetiracetam; AEDs, antiepileptic drugs*.

**Figure 1:**
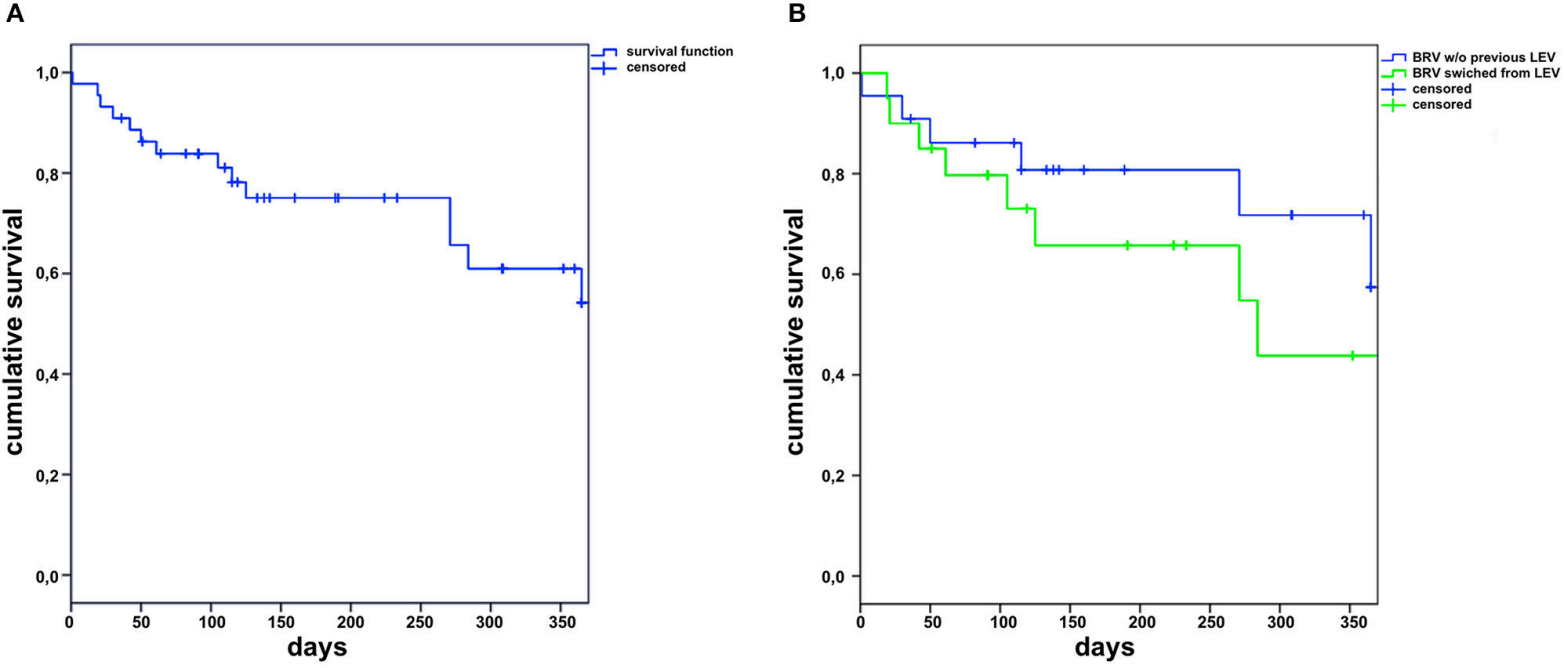
Retention of brivaracetam (BRV) in the complete cohort **(A)** and in patients with (LEV) or without levetiracetam upon start of brivaracetam **(B)** (w/o, without).

A 50% long-term responder rate was apparent in 19 patients (43%), with two (5%) free from seizures for more than 6 months and in nine patients (20%, with one [2%] free from seizures) for more than 12 months. The mean exposure time to BRV was 211 days, ranging from one day to 24 months (median 140 days). The total exposure time to BRV in this study was 310 months. Retention rates were calculated and plotted using the Kaplan–Meier survival curves for all patients (Figure 1A) and depending on the LEV to BRV switch (Figure 1B). No significant difference was observed between patients who started on BRV and those who switched from LEV (log-rank *p*-value: 0.515). At the final follow-up, the daily BRV dose ranged from 50 mg to 300 mg (mean 131.5 ± 86.2 mg, median 150 mg); three patients (6.8%) had a daily dose greater than 200 mg.

### Treatment-emergent adverse events

TEAEs were reported in seven (16%) patients while being treated with BRV. There were six (14%) cases of BAE, one (2 %) of somnolence and one (2%) of bruxism. BAE were observed in four patients that had had BAE while exposed to LEV (*n* = 4/18), while two patients had BAE on BRV who had had no BAE on LEV or were not exposed to LEV in the past (*n* = 2/26, *p* = 0.35). Details are presented in Table 3.

**Table 3:** Characteristics of treatment-emergent adverse events (TEAEs) and their frequency (*n* = 44).

**TEAE under BRV**			TEAE only under LEV^*^	**TEAE under LEV and BRV**
	reported *n*(%)	leading to withdrawal *n*(%)	reported *n*(%)	reported *n*(%)
**Overall**	7 (15.9)	4 (9.1)	18 (40.9)	3 (6.8)
**CNS related**	1 (2.3)	1 (2.3)	5 (11.4)	1 (2.3)
Sedation/somnolence	1 (2.3)	1 (2.3)		
**psychiatric**	6 (13.6)	4 (9.1)	14 (31.8)	2 (4.5)
Irritability	4 (9.1)	2 (4.5)		
Aggression	3 (6.8)	2 (4.5)		
**Other**	1 (2.3)	0 (0.0)	2 (4.5)	1 (2.3)
Bruxism	1 (2.3)	0 (0.0)		

BRV, brivaracetam; LEV, levetiracetam;

* *reported at switch or in the past while exposed to LEV*.

## Discussion

This multicenter retrospective study examined the efficacy of BRV and its tolerability in a cohort of 44 EE patients who represent a severely affected subgroup with usually drug refractory epilepsy and frequent seizures (15, 21). The burden placed on these patients, their caregivers and society makes outcome research in patients with EEs relevant and important. New AEDs including cannabidiol and fenfluramine are being developed for some EE patient subgroups (14–17) and there are few precision medicine approaches for single syndromes leading to EE, such as everolimus in TSC or ketogenic diet in Glut1-deficency. To date, there is only insufficient information on the efficacy and tolerability of BRV in this special subgroup. Data on this cohort, with aggregated 310 month exposure to BRV and follow-ups of up to 24 months, are informative in this respect.

The cohort showed retention rates of 65% at 3 months, 52% at 6 months and 41% at 12 months which is in line with other BRV post-marketing studies (13, 20, 22, 23), and compare with results from other AEDs in frequent use as eslicarbazepine acetate (ESL), LCM, LTG, LEV, perampanel (PER), topiramate (TPM), VPA, and ZNS in patients with focal epilepsies (20, 24–29). Unfortunately, only limited information is available on efficacy and tolerability of AEDs in EE patients. This cohort showed a 50% responder rate of 36% at the 3-month follow-up with additional four patients (9%) being seizure-free. Of these four patients three had been already seizure free during baseline period. The corresponding figures were 43% and 5% at the 6 months follow-up and were 20% and 2% at more than 12 months follow up. These results are in line with other studies using different AEDs in EE (for the most part LGS and DS) including LEV (47%) (30), TPM (40-48%) (31, 32), felbamate (FBM, 50%) (33, 34), ZNS (53 %) (35), and PER (46%) (36) at 3-month follow-up. Other AEDs, and particularly rufinamide (RUF) and cannabidiol (CBD), have been thought promising for EE, and both rendered comparable results with 50% responder rates of 31–48% for RUF (37–39) and 44–50% for CBD (40, 41). Currently, especially the use of CBD as an antiepileptic drug is the subject of some controversy and there is a need for randomized controlled trials (RCT) to verify these findings (42, 43). It has been demonstrated that neurostimulation via an implanted vagal nerve stimulator has similar efficacy with 50% responder rates of 43% (44). Overall, responder rates in EE of ≥50% can be achieved in 30% to 45% of EE patients. While results appear to be similar for different AEDs, there may be differential effects regarding seizure types. PER, for example, may be especially effective for myoclonic or bilateral tonic-clonic seizures (45–47).

No significant difference in efficacy was seen between patients who switched to BRV from LEV and those who either started BRV with LEV treatment at some point in the past or those who had not been treated with LEV before. These findings contrast with other publications that reported lower responder rates with previous exposure to LEV. The difference may result from the small size of our cohort (3, 4), with the limited number of EE patients precluding statistical analysis of possible clinical response predictors. Notwithstanding that, male patients who had a smaller number of previously failed AEDs and a generalized seizure semiology (generalized tonic clonic, myoclonic, absence seizures) trended toward a better response with 50% responder rates exceeding 50%. Rapid titration of BRV (mean 10 days, median 5.5 days) to a mean daily dose of 153.1 mg (median 135 mg) with three patients on a daily dose of more than 200 mg makes under-dosing very unlikely in this study.

BRV was well tolerated in this often severely by BAEs affected subgroup, with only seven patients (16%) reporting TEAE and withdrawal of BRV from only four patients (9%). These findings compare with other post-marketing studies of BRV which showed TEAE in 37–38% (13, 20); the dominant BAEs were symptoms like irritability and aggression (16%). Taking into account of the possibility that this retrospective study may show reporting bias, a comparison with TEAE frequencies seen in trials of RUF (55–70%) (37, 38) and CBD (33–58%) (48, 49), it would seem that tolerance of BRV is good, but careful consideration should be given before using it in patients with a pre-existing intellectual disability (50). The most common reasons for discontinuing BRV were lack of efficacy (23%) and adverse drug events (11%) or both (5%).

Psychobehavioral TEAE were closely followed-up and were present in six patients (14%) while on BRV, leading to discontinuation of BRV in four patients (9%). Psychobehavioral TEAE while on LEV were reported in 14 patients (32%) at switch or in the past while exposed to LEV, details Table 3. Therefore, patients who experience psychobehavioral TEAE associated with LEV might be offered a switch to BRV.

As the study depends on interviews, underreporting of TEAE cannot be ruled out, representing a possible weakness in this study, but all visits and interviews were conducted by epilepsy specialists and documented immediately, minimizing the risk of such bias. Other major limitations of the study are the retrospective chart review and the relatively low number of patients that might lead to unreliable findings and large variability regarding seizure control. The retention rate is a naturalistic functional endpoint encompassing efficacy, quality of life, tolerability, and safety, also no prospective baseline is required (51, 52). Measurement of retention might prove less prone to reporting bias as the prescription of medication is usually well documented.

## Conclusions

BRV is effective and well-tolerated in patients with EE and the pattern of TEAEs compares with other AEDs in frequent use. Efficacy of BRV does not seem to depend on whether patients have previously been exposed to LEV or not. A direct switch from LEV to BRV is feasible for patients with EE. Taken in conjunction with other post-marketing studies on focal or idiopathic generalized epilepsies, it seems that BRV is a reasonable treatment option for patients with epileptic encephalopathies.

## Ethics statement

The study protocol is part of a retrosceptive analysis of efficacy anticonvulsants and was approved by the ethics commitee Frankfurt. Due to the retrosceptive nature of the study, written informed consent was not necessary.

## Author contributions

LW, FR, SS-B, and AS generated the research idea, study design, and concept. LW, AB, FB, FH, II, KMK, SK, RK, GK, LL, GM, KM-S, FvP, PSR, BJS, IS, SS-B, and AS acquired the data. LW, SS-B, and AS analyzed the data and drafted the work. All authors made critical revisions for important intellectual content and interpreted the data. LW, SS-B, and AS wrote the manuscript. All authors approved the final manuscript.

### Conflict of interest statement

AB reports grants from UCB Pharma outside of the reported work and honoraria for speaking engagements from Desitin Arzneimittel, Eisai, and Viropharma. FB reports honoraria for speaking engagements from Desitin and UCB and Eisai. KMK reports personal fees from UCB Pharma, Novartis Pharma, Eisai, and GW Pharmaceuticals as well as grants from the Deutsche Forschungsgemeinschaft and The University of Melbourne. SK reports honoraria for speaking engagements from Desitin and UCB as well as educational grants from ADTech, Desitin Arzneimittel, Eisai, GW, Medtronic, Novartis, Siemens, and UCB. GK obtained honoraria for speaking engagements from Desitin Arzneimittel, Eisai, UCB, Viropharma, Shire, Dibropharma, and Novartis. LL reports industry-funded travel support from UCB Pharma. GM reports industry-funded travel support from Desitin Arzneimittel, Eisai Pharma, and UCB Pharma, as well as speaking honoraria from UCB Pharma. KM-S reports industry-funded travel support from Desitin Pharma, Novartis, Shire, Nutricia, UCB Pharma, and honoraria for speaking engagements Desitin Pharma, Shire, Novartis, Nutricia, Viropharma, Medice, and personal fees from Desitin Arzenimittel. FvP reports industry-funded travel with the support of Desitin Arzneimittel, Eisai Pharma, Bial, and UCB Pharma, honoraria obtained for speaking engagements from Desitin Arzneimittel, Eisai Pharma, Bial, and UCB Pharma, and as part of a speaker's bureau for Desitin Arzneimittel, Eisai Pharma, Bial, and UCB Pharma. BJS reports personal fees and grants from UCB, personal fees as speaking honoraria from Desitin Arzneimittel, Eisai, GW, Hiikma, and Novartis and fees for scientific or medical advice from B.Braun Melsungen. FR reports personal fees from Eisai, grants, and personal fees from UCB, grants and personal fees from Desitin Arzneimittel, Novartis, Medtronic, Cerbomed, ViroPharma, Sandoz, BayerVital, and Shire, research grants from the European Union and grants from Deutsche Forschungsgemeinschaft. SS-B reports personal fees from UCB, Eisai, Desitin Pharma, LivaNova, and Zogenix outside of the submitted work. AS reports personal fees and grants from Desitin Arzneimittel, Eisai, LivaNova, Sage Therapeutics, UCB Pharma, and Zogenix. The remaining authors declare that the research was conducted in the absence of any commercial or financial relationships that could be construed as a potential conflict of interest.
